# Crime and Unemployment

**Published:** 1921-07-02

**Authors:** 


### Crime and Unemployment.

We should like to know what warrant a correspondent has for his suggestion that the increase of crime of all kinds in different parts of the country is due to the enormous mass of unemployment which prevails, and which in turn produces idleness, as well as desperation caused by hunger and fear. In the first place, we have seen no police records which in any way support the statement that crime in this country—apart from the organised and well-recognised political outbreaks of Sinn Fein extremists—has increased in recent months. Indeed, all the indications are the other way. It is a quite remarkable fact that during an unprecedented depression and disturbance of trade, produced or accompanied by a number of acute industrial wage disputes, in which nearly 3,000,000 persons are out of work, the ordinary life of the nation has continued almost without noticeable interruption or disorder.

This preservation of order in circumstances of great temptation or provocation has been commented upon with admiration and surprise by the foreign Press in all parts of the world, and it certainly redounds to the credit of the British people, whose sang-froid and common sense have been put to an exacting test. The explanation seems partly to be this: that there has never been a peroid of trade depression in this country in which out-of-work persons have suffered less real privation. The Prime Minister has been severely criticised for making a statement similar to this to the Dominion Premiers. But what he said was literally true. Not only have the working classes during, and immediately after, the period of war been better paid and (in the post-war period) better nourished than ever before in their history, not only have large numbers of the more far-sighted among them been able to save a good deal of money from their earnings; but the colossal system of unemployment doles (however undesirable from the point of view of national finances and in other aspects as well) has undoubtedly enabled large numbers of persons, who would otherwise have been entirely without the means of subsistence, to feed themselves after a fashion and generally to tide over the crisis. It seems to be just to say that the relatively good physical condition of the masses at the outset of the "slump" has counterbalanced the bad psychological effect of the sudden drop from prosperity to penury, and has thus contributed to keeping them patient, orderly, healthy, and hopeful.

